# Unicentric Castleman Disease of the Mesentery Mimicking a Gastrointestinal Stromal Tumor: A Case Report

**DOI:** 10.7759/cureus.101485

**Published:** 2026-01-13

**Authors:** Ravi Gupta, Jagani Harsha Sai, Vishakha Gupta, Aditya Gaurav, Santhosh Reddy

**Affiliations:** 1 General Surgery, All India Institute of Medical Sciences, Gorakhpur, IND; 2 Pathology, Sanjay Gandhi Postgraduate Institute of Medical Sciences, Lucknow, IND

**Keywords:** castleman disease, hyaline vascular type, lymphoproliferative disorder (lpd), mesenteric neoplasm, right iliac fossa mass

## Abstract

Unicentric Castleman disease (UCD) is a rare benign lymphoproliferative disorder commonly found in the mediastinum and retroperitoneum, with mesenteric involvement being exceptionally uncommon. We present a case of a 21-year-old male with a history of constipation, fatigue, and recurrent right iliac fossa pain for six months. Initial imaging suggested a gastrointestinal stromal tumor (GIST); however, the final diagnosis of UCD hyaline vascular (HV) variant was made based on immunohistochemical (IHC) analysis and histopathological reports. Surgical excision led to complete resolution of symptoms. This unique case makes UCD a differential diagnosis for a right iliac fossa highly vascular mass and underlines the necessity for comprehensive histological and IHC analysis. Successful surgical intervention and exclusion of multicentric Castleman disease (MCD) have resulted in a favorable patient outcome.

## Introduction

Castleman disease (CD) consists of a heterogeneous group of lymphoproliferative disorders, classified into unicentric Castleman disease (UCD) and multicentric Castleman disease (MCD) variants. UCD commonly involves a single lymph node or nodal region and is often asymptomatic or presents with non-specific symptoms [1]. In contrast, MCD presents with constitutional symptoms, and it is a systemic disease involving multiple organs. The mediastinum is the most common site of UCD, accounting for approximately 70% of cases, followed by the intra-abdominal (12%) and cervical regions [1]. Intra-abdominal presentations include retroperitoneal as well as mesenteric locations, of which isolated mesenteric locations are rare and can mimic malignant neoplasms, such as gastrointestinal stromal tumor (GIST), lymphoma, or metastatic disease [2,3]. As per our research using Scopus and PubMed databases till now, 79 mesenteric UCD cases have been reported, out of which four cases belong to the large bowel mesentery. In these four cases, only one case reported the right mesocolic location, which shows the peculiar nature of this case report. So UCD becomes one of the rare differential of enhancing mass lesions arising from the right mesocolon. This report discusses a case of mesenteric UCD diagnosed preoperatively as GIST on imaging, which surprisingly turned out to be UCD on the final histopathological report. This case emphasizes the need to consider uncommon differential diagnoses when evaluating patients with localized abdominal masses and highlights the diagnostic difficulties arising from the similar clinical features of CD and GIST.

This study was previously published as an e-poster for the same case report, which was presented at CME (Continuing Medical Education) Surgery: 2024 at the All India Institute of Medical Sciences, New Delhi, on September 5, 2024.

## Case presentation

A 21-year-old male presented with a history of constipation, fatigue, and recurrent right iliac fossa pain for six months. Notably, he had no fever, weight loss, or other constitutional symptoms. On examination, there were no signs of hepatosplenomegaly, anemia, or generalized lymphadenopathy. Both testicles were normal on examination, and no abdominal mass was palpable. Laboratory investigations revealed a normal platelet count (280×10³/μL), hemoglobin (14.5 g/dL), and albumin (4.1 g/dL), and negative serology for human immunodeficiency virus/hepatitis B virus/hepatitis C virus (Table 1).

**Table 1:** Laboratory investigations at the time of presentation. SGOT: serum glutamic oxaloacetic transaminase; SGPT: serum glutamic pyruvic transaminase; HBsAg: hepatitis B surface antigen; HCV: hepatitis C virus

Parameters	Patient value	Reference range
Hemoglobin (g/dL)	14.5	12.6-17.1
Total leukocyte count (×10³/µL)	5.40	4.0-11.0
Platelet count (×10³/µL)	280	150-400
Serum albumin (g/dL)	4.1	3.2-4.5
Total protein (g/dL)	8.4	6.4-8.3
SGOT (U/L)	30.9	0-40
SGPT (U/L)	22.2	0-45
Serum creatinine (mg/dL)	0.55	0.5-1.5
HIV/HBsAg/HCV	Non-reactive	Non-reactive

**Figure 1:**
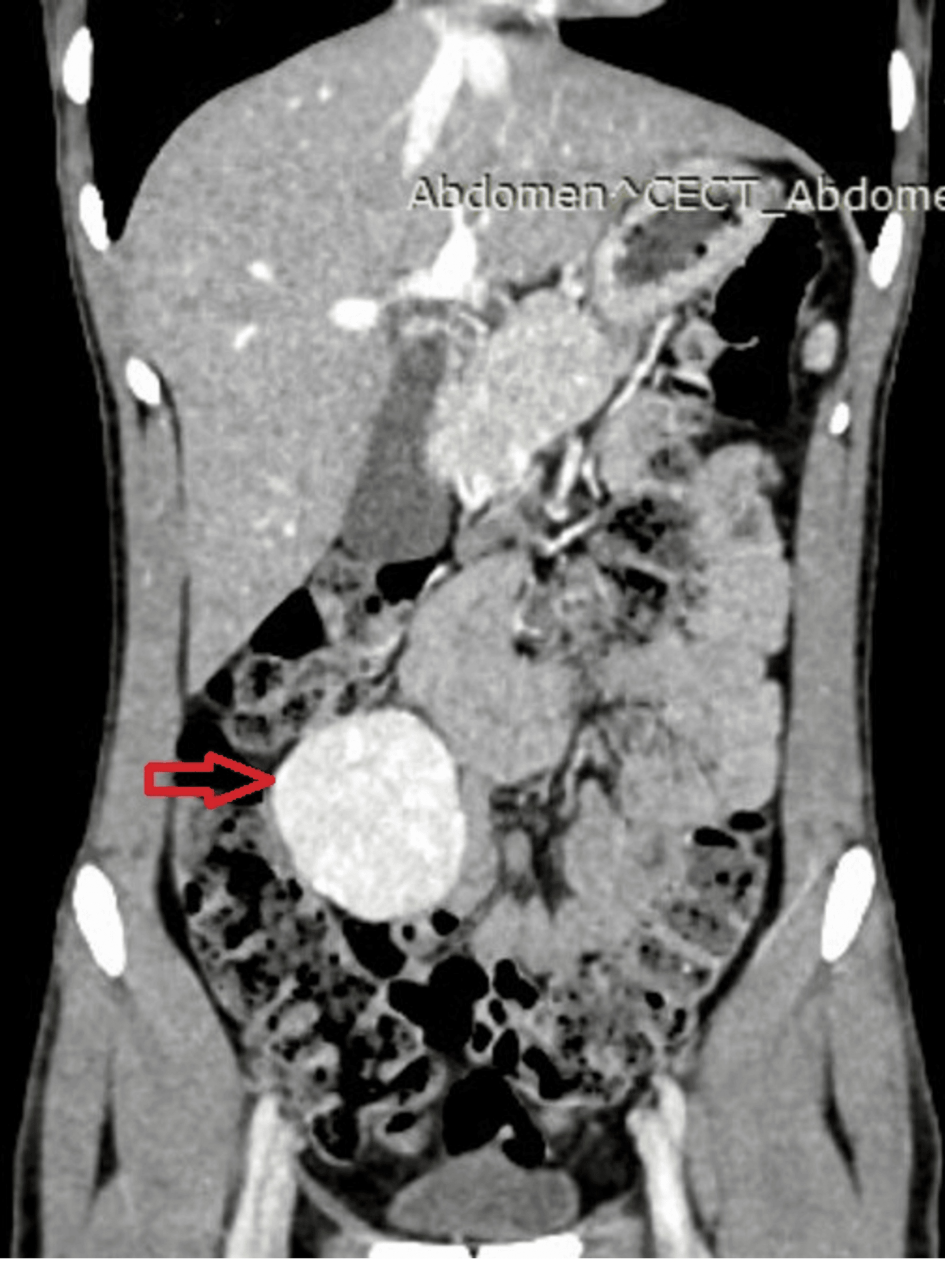
Coronal CECT abdomen showing intensely enhancing mesenteric mass in right iliac fossa (4.3×3.2 cm, red arrow) abutting small bowel loops. CECT: contrast-enhanced computed tomography

**Figure 2:**
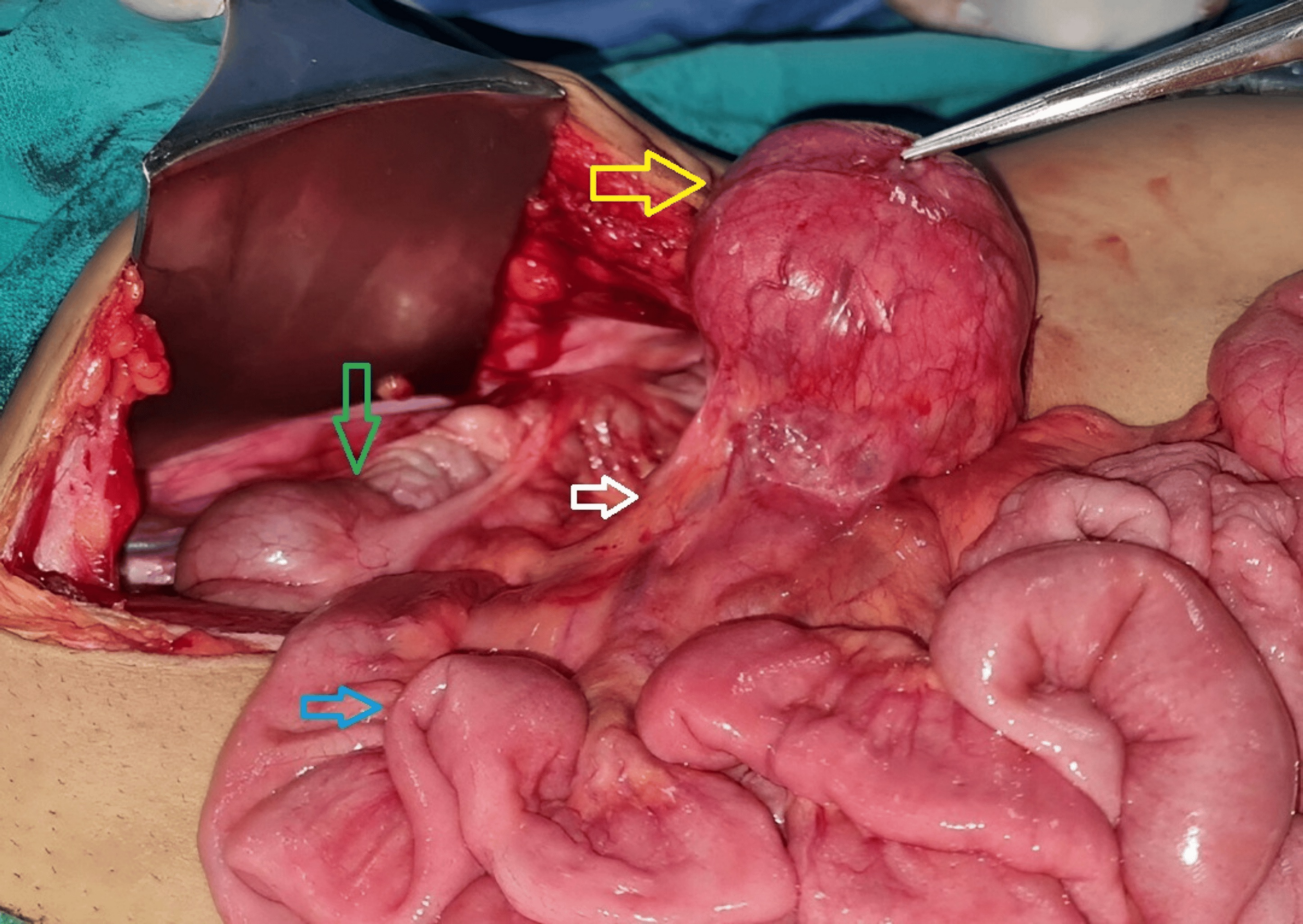
Intra-operative picture (craniocaudal view) showing pedunculated vascular mass (yellow arrow), ileocolic vascular pedicle (white arrow), ileum (blue arrow), and ascending colon (green arrow).

**Figure 3:**
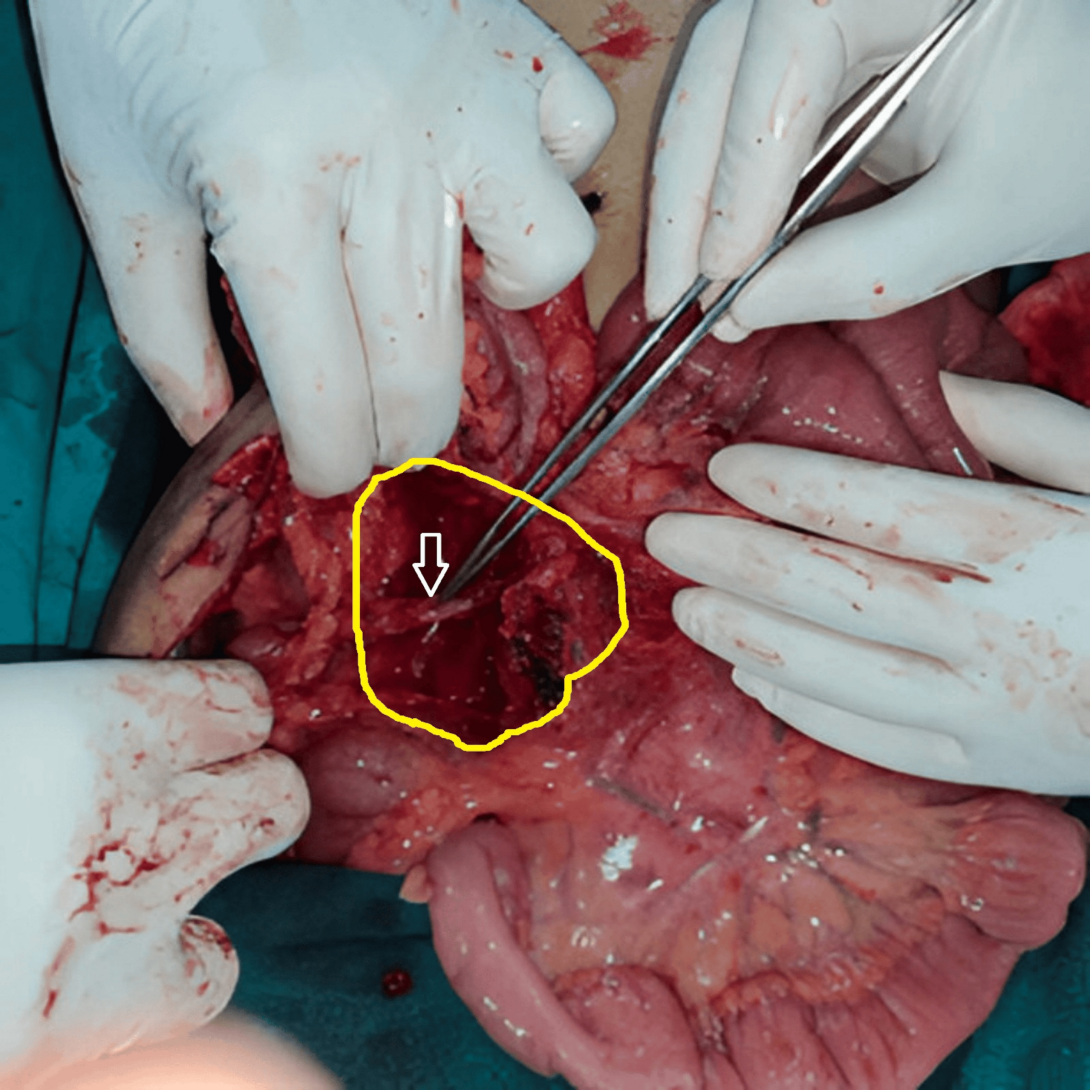
Post-resection picture showing preserved ileocolic vessels (white arrow), tumor bed (yellow line) with clear resection margins.

**Figure 4:**
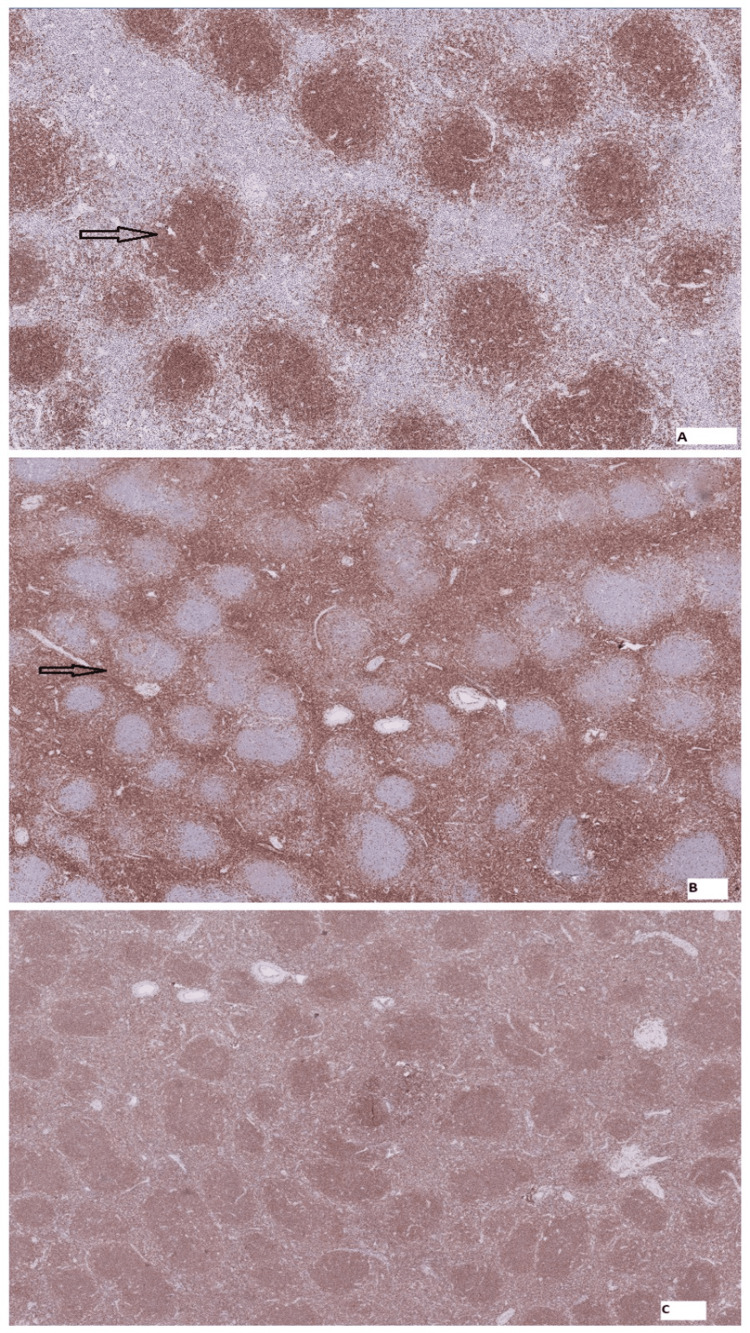
Immunohistochemistry picture (20x) showing CD20, CD3, and BCL2 positive. Panel A shows germinal centers strongly positive for CD20 (black arrow). Panel B shows CD3‑positive T‑cells predominantly localized in the parafollicular/interfollicular areas (black arrow). Panel C shows diffuse BCL2 positivity in mantle zone B‑cells (black arrow).

A contrast-enhanced computed tomography (CECT) whole abdomen showed a clearly demarcating enhancing mass in the right iliac fossa, which was initially suggestive of a GIST, due to its location and enhancement pattern (Figure 1). To further assess the lesion for possible malignancy, positron emission tomography-computed tomography (PET-CT) was performed, which demonstrated a solitary fluorodeoxyglucose (FDG)-avid mesenteric lesion (SUVmax 7.56) without evidence of distant metastasis or multicentric disease. Owing to the loss of the original documentation, the PET-CT images could not be included. Based on these findings, GIST was considered the leading diagnosis, and a surgical excision plan was developed. The patient subsequently underwent resection of the mesenteric mass (Figures 2, 3). A midline laparotomy was performed, with wide local excision of a pedunculated mass arising from the right mesocolon, preserving the ileocolic artery and vein, which further helped in preservation of the vascularity of the cecum and ascending colon. During surgery, the mass was noted to be highly vascular, which initially supported the diagnosis of GIST. However, histopathological examination unexpectedly revealed complete effacement of the lymph node architecture, replaced by multiple, similarly sized follicles involving both the cortex and medulla. The germinal centers comprised centrocytes and centroblasts, a histopathological pattern consistent with UCD, specifically the hyaline vascular (HV) variant, rather than GIST, which typically demonstrates spindle-shaped cells with epithelioid features. So the immunohistochemical (IHC) staining was done related to CD, which showed positivity for CD20, CD43, CD3, CD5, Pax5, BCL2, and CD79a, confirming the diagnosis of CD (Figure 4, panels A-C). Following surgery, the patient recovered well, and the post-operative event remained uneventful. At the 18-month follow-up, the patient was asymptomatic. Follow-up clinical examination and imaging (PET-CT) revealed no evidence of recurrence. This case underscores the diagnostic challenges posed by rare conditions like CD and the need for a thorough investigation to arrive at the correct diagnosis.

## Discussion

CD represents a rare, heterogeneous group of lymphoproliferative disorders first characterized by Castleman et al. in 1956 [4]. It is traditionally classified by its clinical extent into UCD, involving a single lymph node or nodal region, and MCD, which involves multiple lymph node stations and often presents with systemic inflammatory symptoms [5,6]. As mesenteric UCD is rare, this condition is frequently mistaken for a malignant growth due to its similar presentation. In our case, the patient presented with recurrent right iliac fossa pain, and on imaging, a hypervascular lesion was identified, leading to a common differential diagnosis of mesenteric mass that includes GIST, lymphoma, neurogenic tumors, and metastatic disease.

On a histopathological basis, the HV variant is the most common subtype in UCD (80-90%) [7]. It is characterized by atrophic or "regressed" germinal centers with expanded mantle zones containing small B-lymphocytes arranged concentrically in an "onion-skin" pattern [8]. A hallmark feature is the presence of a penetrating "lollipop-like" hyalinized blood vessel [6]. IHC analysis is essential for confirmation and to exclude mimics. While CD20-positive B cells dominate the mantle zone, CD3-positive T cells are typically found in the paracortical and interfollicular regions [9]. Furthermore, markers, such as CD21 or CD23, are used to identify the expanded follicular dendritic cell (FDC) networks characteristic of the HV variant [8].

HV variant was seen in this case, and it was characterized by prominent follicular hyperplasia, small germinal centers comprised of centrocytes and centroblasts with an increased vascular component [10]. IHC markers, including CD20 and CD79a, aid in distinguishing UCD from lymphomas and other malignancies. It is essential to consider UCD in the differential diagnosis of well-defined, highly vascularized mesenteric masses to avoid misdiagnosis. In our case, IHC staining showed positivity for CD20, CD43, CD3, CD5, Pax5, BCL2, and CD79a, confirming the diagnosis of UCD. Other IHC markers like LANA-1 and HHV-8 staining are more helpful in cases of MCD [11]. Surgical excision remains the gold standard for managing UCD and is curative in most cases [12]. In our case, we did an ileocolic vessel-sparing wide local excision of the mass, considering the pre-operative diagnosis of GIST, which led to the preservation of the corresponding small and large bowel vascularity. Unlike MCD, which requires systemic therapy, UCD generally does not recur post-excision. Complete resection provides symptom relief and prevents potential complications such as mass effect and secondary compression of surrounding structures. Post-operative follow-up is necessary, although recurrence is rare in unicentric disease [12]. Our case has an 18-month follow-up with no evidence suggestive of local recurrence and distant metastasis. Recent case reports and reviews highlight that a multimodal diagnostic approach, which includes cross-sectional imaging and detailed histopathological evaluation with targeted immunohistochemistry, is essential. These are essential to accurately diagnose CD and to distinguish unicentric from multicentric variants and to exclude mimickers, such as GIST, lymphoma, and other hypervascular mesenteric tumors [3]. PET-CT demonstrated a solitary right iliac fossa FDG-avid lesion (SUVmax 7.56) without multifocal disease, favoring UCD over MCD. HV variant typically exhibits low-moderate uptake (SUVmax 4.1) due to vascular stroma and plasma cell variant (SUVmax 5.3) due to inflammatory activity. GIST usually shows higher uptake (SUVmax ≥5.68) [13,14]. In this case, the lesion has a higher avidity (SUVmax 7.56) with a mesenteric location, keeping GIST as a differential. Hence, definitive diagnosis requires histopathological analysis. PET-CT remains valuable for post-operative surveillance to detect recurrence [14].

## Conclusions

UCD deserves consideration during diagnostic workup of mesenteric tumors, particularly when imaging features suggest a well-circumscribed, hypervascular mass. Histopathological and IHC evaluation is essential for accurate diagnosis. Complete surgical excision is both diagnostic and curative, with an excellent prognosis. This case shows the vital role of multidisciplinary collaboration in diagnosing and managing rare abdominal pathologies.
